# The survey of serum retinol of the children aged 0~4 years in Zhejiang Province, China

**DOI:** 10.1186/1471-2458-7-264

**Published:** 2007-09-25

**Authors:** Rongwang Yang, Rong Li, Shujiong Mao, Liying Sun, Xinwen Huang, Chai Ji, Zhiwei Zhu, Lingling Wu, Yufeng Qin, Zhengyan Zhao

**Affiliations:** 1 Department of Child Healthcare, the Children's Hospital Affiliated to Zhejiang University School of Medicine, Hangzhou, China; 2 Department of Child Healthcare, Hangzhou Women and Children's Hospital, Hangzhou, China

## Abstract

**Background:**

Vitamin A can have a positive impact on growth and development of children, but vitamin A deficiency (VAD) was found to be a public health problem in Zhejiang Province, China in 1998. There have been no studies on this topic in Zhejiang Province recently. This study was designed to evaluate the serum retinol levels of children aged 0~4 years in Zhejiang Province, southeast China. This epidemiological data will help design supplementation strategies for vitamin A in high-risk groups and improve their vitamin A status.

**Methods:**

Children were randomly recruited for this study using a stratified sampling method. A blood sample was collected from each child. Assessment included C-reactive protein (CRP), serum retinol was measured with HPLC and a questionnaire completed providing for family information and nutritional status. Logistic regression analysis was used to evaluate the risk factors for VAD in children.

**Results:**

A group of 357 subjects aged 1 day to 4 years were recruited. The mean plasma retinol concentration was 1.653 (sd 0.47) μmol/L. There were 3.08% (11/357) of children affected with VAD, and 7.28% (26/357) of children had low vitamin A status, but none of the children showed any clinical symptoms of VAD. There was no significant difference in the levels of plasma retinol and the incidence rate of VAD between male and female children. Multivariate logistic regression analysis showed that living in urban region, having parents with good education and taking vitamin A capsule regularly prevented children from VAD, whereas being young (less than 2 years old) was a risk factor.

**Conclusion:**

Low vitamin A status remains a nutritional problem in Zhejiang Province. The high-risk group in this study were young, dwelled in rural regions, had parents with poor education and did not take a regular vitamin A containing supplement.

## Background

Vitamin A is one of most important nutrients which are essential to all, especially children and pregnant women [1,2]. It plays an important role in cellular differentiation, which is critical in growth, reproduction and immune response. Children with VAD have a tendency to be more affected by infection [3,4] and xerophthalmia [5]. In addition, VAD is associated with increased mortality in young children [6-9]. Improving the vitamin A status of young children can reduce child death rates by 20–50% [10,11], and enhance both cellular and humoral immune responses in animals as well as in humans [12,13].

Nowadays, severe VAD has been controlled efficiently with the efforts of the World Health Organization (WHO), the United Nations Children's Fund (UNICEF) and the International Vitamin A Consultative Group (IVACG), etc. [8,14]. But in many countries, especially those developing, VAD is still a public health problem[15,16]. It was estimated that more than 127 million preschool children are affected by VAD and 4.4 million with xerophthalmia [17] in the world. Most of them were from Africa and Southeast Asia because of an insufficiently varied diet, little food with abundant vitamin A, poor maternal education and inadequate hygiene [17,18]. Epidemiological data on VAD can be useful in planning, designing, and targeting interventions.

China is a developing country. Jiang et al. [19] found that the prevalence of VAD in children under 6 years was 12.2% in 2006, which suggested that VAD was still a public health problem in China. In Zhejiang Province, an investigation of VAD carried in 1998[20] showed that the incidence of VAD was 7.87% in children aged 0~6 years old. Eight years later, with the support of the Working Committee on Children and Women of Zhejiang Province, we performed an interview to get further epidemiological data of VAD in 2006. The objective of this study was to evaluate the serum retinol levels of children in Zhejiang Province. These data would be important in drawing the policy to prevent VAD and improving the vitamin A status in Zhejiang Province and other districts in China. In addition, the risk factors for low serum retinol will allow community health program policymakers to perfect strategies for improving vitamin A status in the at-risk communities.

## Methods

### Design and Sampling

The survey was performed in Zhejiang province from April to November, 2006. Zhejiang is located in the southern part of the Yangtze River Delta on the southeast coast of China, bordering East China Sea. In this study Zhejiang was divided into 3 parts according to geographic conditions, including mountains, plains and littoral area. Two-stage sampling method was used to reach the children. In the first stage, a sampling method was used to identify 2 counties in each part. In the second stage, a systematic random sampling method was used to identify 75 children aged from 0 day to 4 years 11 months 30 days old per county, which were accorded with the criteria. All children in the household selected were included in the study if verbal consents were provided by the parents/caregivers.

Child age in months was assessed as a continuous variable, and a categorical variable was constructed yielding five categories: (1) 0–5 months, (2) 6–11 months, (3) 12–23 months, (4) 24–35 months, (5) 36–47 months, and (6) 48–59 months. Child sex was ordinal (1) male, (2) female.

### Data Collection

A pilot structured questionnaire was first tested and updated in a survey with similar settings and then administered face-to-face with the parents/caregivers. Trained non-medical university students gathered data to minimize bias, training of interviewers emphasized proper and random identification of respondents and questionnaire administration.

Interviewers collected the information on the educational level of parents, socioeconomic level, dwelling district, history of feeding, child's appetite, quantity of foodstuff rich in vitamin A, whether provided with capsule of vitamin A, illness history for the previous 2-week period. Child's age was obtained through birth certificates, health cards. The questionnaire was based on the demographic and health survey questions to avoid separate validity and reliability checks of the data collection instrument.

### Clinical examination

All children were examined by the doctor of the survey team for ocular signs of VAD, such as conjunctival xerosis, Bitot's spots, corneal xerosis, etc

### Blood sampling and biochemical measurements

A blood sample (5 ml) was collected by venepuncture of an antecubital vein or jugular vein, and the volume of sample was about 1 ml for children whose ages were less than 1 year old after verbal consents were obtained from the parents/caregivers. The specimens were collected in red-top serum separator tubes and immediately wrapped in aluminum foil, continuously shielded from light, and stored at 4°C until centrifugation (200 g × 10 min, room temperature). Aliquots of serum were made and immediately frozen at -10°C, then shipped to Hangzhou City (capital of Zhejiang Province) in a portable ice box filled with solid carbon dioxide. Then all were kept frozen at -70°C until analyses.

The plasma retinol concentration was determined by reversed-phase high-performance liquid chromatography (HPLC) according to the method of Bieri et al. [21]. All extraction and HPLC procedures were carried out under reduced light in order to prevent oxidation of the compounds. A total of 0.2 ml serum was added to 0.1 ml standard solution of retinyl acetate, and was depolarized by 0.1 ml ethanol. Serum vitamin A was extracted with hexane (0.6 ml) and a portion of the sample (0.4 ml) evaporated to dryness under nitrogen and re-dissolved in 0.1 ml mobile phase(methanol: water, 95:5 by vol.), and injected into a C_18_, reversed phase HPLC column (5 mm particle size; Beckman Instruments, Inc.). The flow rate of mobile phase was 1 ml/min. Retinol was detected by monitoring the absorption at 325 nm in a Beckman ultraviolet detector. The vitamin A concentration was quantified to the peak area of the internal retinyl acetate standard. All retinol analyses were performed by the same individual. For serum retinol, the within-assay and between assay CVs were 3% and 8%, respectively.

CRP concentration was measured by using a latex turbidimetric immunoassay method with a commercial kit (CRP-Latex Seiken; Denka Seiken Co., Ltd, Tokyo, Japan). The intra-assay coefficient of variation for CRP was 1.2%. CRP concentration was used as indicators of the presence of a possible infection or inflammation. CRP concentration ≥ 8.0 mg/L was considered abnormal.

### Criteria

The prevalence of VAD was assessed according to the recommendations of WHO and expert committee reports [22]. Xerophthalmia was diagnosed if any clinical signs of vitamin A deficiency were exhibited in one eye or both eyes; Children with plasma retinol concentrations < 0.70 μmol/L(< 20 μg/dL) and between 0.70 and 1.05 μmol/L(between 20 and 30 μg/dL) were characterized as being VAD or having low vitamin A status, respectively. But if the children with a fever (> 38.0°C) during the previous 2 weeks enrolled in the study or a C-reactive protein concentration ≥ 8.0 mg/L were considered having inflammation recently and excluded from the study.

### Statistical analysis

All statistical analyses were done with blinding maintained and *P *value of less than 0.05 was regarded as statistically significant for all statistical tests. Data were entered in Excel software, and analyzed in SPSS (version 13.0) software package. The mean serum retinol levels difference between boys and girls was assessed by the student t-test. Chi-square analyses were performed to compare the incidence of VAD between boys and girls. Several categories of independent variables considered to affect serum retinol status were examined: (1) nutrition-related factors (anthropometric values, frequency of breastfeeding, duration of exclusive breast feeding, etc.), and (2) demographic/socioeconomic indicators (parents' age and years of formal education, mother's career, dwelling type, etc.). Logistic regression models were developed to assess risk factors for VAD using covariates that were statistically significant at a level of P = 0.10 in the univariate analyses.

### Ethics

Approval of the study was granted by Zhejiang University Faculty of Medicine Ethics and Research committee and the Regional Committee for Medical Research Ethics, Zhejiang Province, P.R.C.

## Results

A total of 450 children were eligible to be enrolled into the study, 29 of whose parents (6.4%) refused to let their children participate. Blood samples were available from 389 of the 421 (92.4%) for the assessment of vitamin A and CRP status. CRP levels of 32 children were more than 8 mg/L, and these children were excluded from the study. Therefore, data of serum retinol concentrations were available for 84.8% (357/421) children enrolled in this study, including 188 boys and 169 girls. There were 189 children from rural region and 168 from urban region.

**Figure 1:**
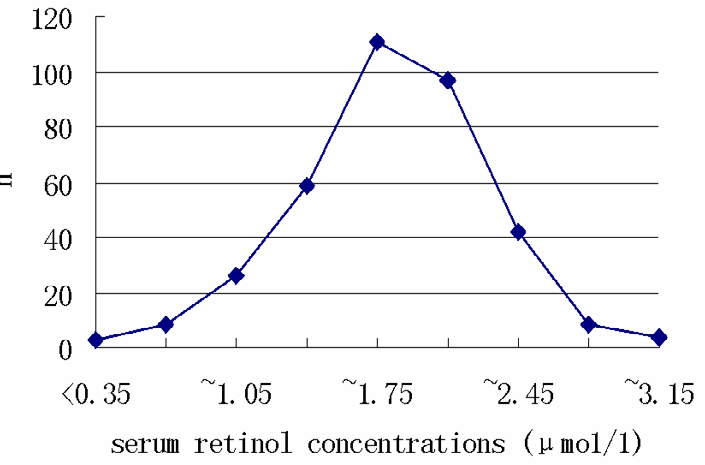
Frequency distribution curves per serum retinol concentration range.

The mean serum retinol concentration for the 357 children was 1.65 ± 0.47 μmol/L (range: 0.147–3.08 μmol/L). The frequency distribution of concentrations is provided in Figure 1. Approximately 89.6% (320) of the children had serum retinol concentrations in the normal range. Serum retinol levels by gender and residential area are provided in Table 1. There were no statistically significant differences in mean serum retinol and numbers of children with serum retinol < 1.05 μmol/L between boys and girls.

**Table 1:** Comparison of different characteristics with respect to vitamin A status of children

Characteristics		Mean serum retinol Level (μmol/L)	t-value	P- value
Gender	Male(n = 188)	1.66 ± 0.45	0.318	0.751
	Female(n = 169)	1.65 ± 0.49		
Residential regions	Urban(n = 189)	1.66 ± 0.49	0.078	0.938
	Rural(n = 168)	1.63 ± 0.43		

In this survey, there were 11 children affected with VAD, 26 children with low vitamin A status, but there were no children with any clinical symptoms, such as Bitot's spots, conjunctival xerosis, corneal lesions. The incidence of VAD was 3.08% (11/357), and the percentage of children with low Vitamin A status was 7.28% (26/357). The table 2 showed that the ratio of children with VAD and low vitamin A status were higher in age groups less than 2 years old. There was no statistically significant difference between boys and girls with VAD and low vitamin A status. There was a tendency for age groups less than 2 years old to have lower serum retinol level and higher VAD incidence. (Table 2)

**Table 2:** Serum retinol levels and VAD in every age group

Age group (months)	Number (n)	Serum retinol(μmol/L)	Clinical signs (%)	VAD (%)	Low Vitamin A status(%)
0–5	38	1. 51 ± 0. 68	0(0)	6(15. 8)	1(2. 6)
6–11	35	1. 56 ± 0. 51	0(0)	3(8. 6)	6(17. 1)
12–23	70	1. 67 ± 0. 47	0(0)	2(2. 9)	9(12. 9)
24–35	68	1. 63 ± 0. 42	0(0)	0(0)	3(4. 4)
36–47	72	1. 66 ± 0. 35	0(0)	0(0)	3(4. 2)
48–59	74	1. 68 ± 0. 45	0(0)	0(0)	4(5. 4)
t ot al	357	1. 65 ± 0. 47	0(0)	11(3. 08)	26(7. 28)

Table 3 shows that 4 risk factors were associated with VAD according to univariate analysis. Younger age in month, residential rural region, less average education years of parents and irregularly taking vitamin A capsule increased the risk of VAD in children. In the groups of children who had one of these risk factors or their combination, a significantly increased risk of VAD was found.

**Table 3:** Risk factors for children with VAD and low serum Vitamin A status (univariate analysis) (n = 357)

Variable	Odds ratio	95% confidence interval	P-value
Age (per unit increase in month)	0.76	0.38–1.07	< 0.001
Residential regions(urban *vs *rural)	4.65	1.52–8.74	< 0.001
Average education years of parents (per unit increase in year of education)	0.84	0.75–0.94	0.003
Oral administration of vitamin A capsule(no *vs *yes)	0.42	0.18–1.12	0.005

Multivariate analysis (Table 4) demonstrated that each month of children growing was associated with a 36% decrease in odds ratio of VAD. Children dwelling in rural regions were nearly 4.5 times as likely to be VAD as those in urban regions. In contrast, parents' education was inversely associated with VAD. Each year of education experienced by parents reduced 23% in the odds ratio of VAD. The children taking vitamin A regularly were less possible in the risk of being suffered from VAD.

**Table 4:** Risk factors for children with VAD and low serum Vitamin A status (multivariate logistic regression analysis)(n = 357)

Variable	Odds ratio	95% confidence interval	P-value
Age (per unit increase in month)	0.64	0.45–1.11	< 0.001
Residential regions(urban *vs *rural)	4.52	1.48–8.92	0.004
Average education years of parents (per unit increase in year of education)	0.77	0.67–0.89	< 0.001
Oral administration of vitamin A capsule(no *vs *yes)	0.35	0.11–1.01	0.007

## Discussion

Vitamin A is important for the eyes and skin, and for normal growth[23,24]. It is essential for the maintenance of healthy epithelial tissue of the skin, eyes, respiratory system, gastro-intestinal and urinary tracts[25,26]. VAD affects the growth and development of children greatly, but VAD has been given little attention in that severe VAD with clinical signs such as xerophthalmia and night blindness are rarely seen recently. However, some research[3,11,25,27-30] have shown that VAD even at low vitamin A status might still have negative effects on health and an increase in morbidity and mortality among children and that vitamin A supplementation reduces the frequency of severe and lethal illness[11,31-34]. It is important, therefore, to assess the status of vitamin A of the children who are in risk of VAD before drawing and carrying out an intervention strategy.

The result of a low prevalence of VAD among children under 4 years old without xerophthalmia and Bitot's spots was a significant finding of this study. Low vitamin A status was more popular. It is difficult to compare our results with prior surveys because of different age strata and different vitamin A assays. However, the survey of 1998 in Zhejiang Province [20], indicated a prevalence of 7.8% for VAD, which decreased to 3.08% in 2006. A summary of the characteristics of the two studies is shown in Table 5. From 1990 to 2006, beside national surveys[19,35], a number of studies [36-38] have also investigated the prevalence of VAD in China, which are showed in Table 6. Studies in Inner Mongolia[39] (North China), Shandong Province[40] (East China), Tibet[41] (West China) showed incidences of VAD in children of 18.4%, 7.8% and 8.4%, respectively. These are all higher than the present survey. The prevalence of VAD in Zhejiang Province was also lower than the findings from Africa [42] and Southeast Asia [43,44].

**Table 5:** Characteristics of investigations in Zhejiang Province in 1998 and 2006

Survey date	Sample resource	Sample size(n)	Male/ female	Detection method	Serum retinol(μmol/L)	No. Of VAD(%)
October 1998	13 counties	1842	968/874	fluorescence	1.62 ± 0.92	145(7.8)
April2006	6 counties	357	188/169	HPLC-UV	1.65 ± 0.47	11(3.08)

Incidence of VAD, χ^2 ^= 10.41, P = 0.001

**Table 6:** Surveys of serum vitamin A status in Provinces of China

Survey date	Sample resource	Sample size	Age group (y)	Detection method	Serum retinol (μmol/L)	No. Of VAD(%)	No. of low vitamin A status(%)
October 2006	Zhejiang Province	357	0~4	HPLC-UV	1.65 ± 0.47	11(3.08)	26(7.28)
October 2002	Fujiang Province [36]	3185	0~5	fluoresce nce	--	200(6.3)	838(26.3)
June 2000	Liaoning Province [37]	10,784	0~6	fluoresce nce	1.20 ± 0.44	1132(10.5)	3570(33.1)
April 2000	Jiangsu Province [38]	1170	0~5	fluoresce nce	--	154(13.2)	--
March 2000	Shandong Province	613	0~5	fluoresce nce	1.1	48(7.8)	202(32.9)
December 1999	Inner Mongolia	636	0~5	fluoresce nce	0.96	117(18.4)	308(48.4)
December 1999	Tibet	1257	0~6	Fluoresce nce	1.09 ± 0.32	106(8.4)	483(38.4)
December1999	China	8669	0~6	fluoresce nce	1.06 ± 0.33	1018(11.7)	3396(39.2)

The causes of the low prevalence of VAD detected in Zhejiang Province have not yet been studied. The most probably hypothesis from the questionnaire data is that the children consume a large amount of vitamin A-rich foods. Probable causes were as following: 1). great improvement in economics and urbanization recently has contributed to an overall improvement in living standard similar almost to that of a fairly well-off society, which resulted in ample food; 2). diverse information on nutrition and feeding from media and news paper made the study population attach more importance to health and children's nutrition; 3). dissemination of information about the problem in the media and the training of health professionals so that they would recognize the problem and be able to instruct the parents about a balanced diet containing foods rich in vitamin A. In addition, former studies [41,45,46] aiming at the identification of the risk factors for VAD in other districts are measures that could be adopted to fight VAD in Zhejiang Province. Furthermore, oral administration of vitamin A capsule (including Vitamin A 1500 IU or 2000IU every one) everyday was performed in China to prevent VAD in the children under 2 years old.

No clinical sign of VAD was found in the surveyed populations. However, both questionnaire data and biochemical criteria revealed the prevalence of low vitamin A status was more prevalent in the past.

Our logistic regression analyses showed that younger children in age experienced a greater risk of VAD than did older children. In general, all infants are born with very limited liver vitamin A stores, about 6 μmol total stores, or 0.04 ìmol/g(liver), which is less than a 2-wk supply[47]. Healthy, well-nourished infants rapidly accumulate liver vitamin A stores, achieving an adult concentration of 0.07ìmol/g(liver) (or about 20 ìmol total stores) by 6 months of age. Liver stores of vitamin A accumulate as a function of the absorbed intake, basal requirements and catabolic losses of the vitamin. Dietary intake depends on the volume and vitamin A content of breast milk, foods and supplements consumed and the percentage absorbed from each source [48]. Intaking fewer types of food, reducing absorption, and more illness increasing catabolic rate in younger children contribute to lower serum vitamin A status. An optimal strategy is to prevent children younger than 2 years old from being affected by VAD.

Residential region was also a risk factor for vitamin A deficiency. Results from other studies [41,46] in China indicate that it's more convenient for the ones dwelling in urban regions to get nutritional information, intake more food with vitamin A, and receive more medical instruction. Also the children dwelling in rural area were affected by dietary habits and disease patterns that similarly adversely affected protein energy and vitamin A status [49]. There was experimental and clinical evidence showing that low protein status could impair the production of retinol-binding protein in the liver [50]. Protein energy malnutrition usually emerged in the children of rural area in China. Therefore, advocation of consuming food with vitamin A and the prevention of chronic malnutrition are likely to improve serum retinol concentrations in countryside.

Parents' education was negatively associated with VAD. Parents with a higher educational level may receive more knowledge on nutrition. Encouraging boys and girls to study during the school age could potentially improve the vitamin A status of their children. Furthermore, development of nutrition education programs targeting parents of child-bearing age may help to improve the vitamin A status in women and their children in addition to the general health and well-being.

We also found that the children taking vitamin A capsule(containing 1500IU and 2000IU vitamin A for children less than 1 year and 1~2 years, respectively) daily had less with VAD. The amount of vitamin A in these capsules could almost meet the criteria of the RDA. An additional suggestion for parents is that their children under 2 years old should take a vitamin A capsule everyday.

These results might be affected by a number of biases. However, selection biases were actually controlled by using a representative sample of children randomly drawn from the different districts of Zhejiang. To minimize information biases, the same physician performed all clinical examinations in the Children's Hospital. In addition, single experienced technician read all the histological tests. The dietary questionnaire was given in the local language, collected by trained non-medical university students and dealt with by an experienced nurse. Similarly, the HLPC assays were all processed in the same laboratory. However, absence of the external standard was a limitation for the study. Because of the limitation in sample size and methodology, possible clinical signs and the prevalence of VAD might not have been revealed. We should enlarge the size to get more accurate epidemiological data of VAD in Zhejiang Province.

## Conclusion

Low vitamin A status is a nutritional problem in Zhejiang Province. The high-risk group is the children being younger, dwelling in rural regions, having parents with poor education or not taking vitamin A capsule regularly.

## Competing interests

The author(s) declare that they have no competing interests.

## Authors' contributions

RY, RL and ZZ were responsible for the conception and design of the study. RY and SM performed the data analysis. All authors participated in interpretation of the findings. RY and SM drafted the manuscript. RL and ZZ revised and commented on the draft and all authors read and approved the final version of the paper. All authors confirm that the content has not been published elsewhere and does not overlap or duplicate their published work.

## Pre-publication history

The pre-publication history for this paper can be accessed here:


